# Cutaneous lymphangioma circumscriptum

**DOI:** 10.11604/pamj.2023.45.46.39047

**Published:** 2023-05-18

**Authors:** Adithya Kiran, Asritha Komandla

**Affiliations:** 1 Department of Pediatrics, Jawaharlal Nehru Medical College, Acharya Vinoba Bhave Rural Hospital, Datta Meghe Institute of Medical Sciences, Sawangi, Wardha, Maharashtra, India

**Keywords:** Lymphangioma, frogspawn, dermoscopy, excision

## Image in medicine

An 11-year-old boy presented to the Out Patient Department (OPD) with a slow growing, asymptomatic lesion on his left shoulder region. The lesion was present since birth and had gradually increased in size over time. The patient reported no pain or discomfort associated with the mass. Physical examination revealed erythematous clusters of translucent intact vesicles, containing clear fluid of varying sizes (2 mm - 5 mm) with few crusts on the left shoulder (A). Few vesicles had ruptured, however there was no evidence of local inflammation or cellulitis. Few of these channels were filled with clear fluid (B). Dermoscopy revealed frogspawn like lesions with white lined structures (C). Hence, the diagnosis of lymphangioma circumscriptum was made. The patient was referred to a plastic surgeon for excision of the lesion. Post-operative healing was uncomplicated, and the patient made a full recovery and was discharged and monitored for recurrence. Cutaneous lymphangiomas are a rare congenital malformation of the superficial cutaneous lymphatic ducts. Lymphangioma circumscriptum (LC) is a lymphatic malformation that is composed of superficial lymphatic vessels, microcysts on the skin or mucous membrane. They occur as a result of an error in the morphogenesis of the lymphatic vessels, leading to a disorder of lymph drainage. It is clinically defined by a clustering of vesicles with varying content in blood. The symptoms may include pruritus, pain, burning, lymphatic drainage, infection, and aesthetic concerns. Although dermoscopy plays a major role in the diagnosis, histology remains the gold standard for diagnosis.

**Figure 1:**
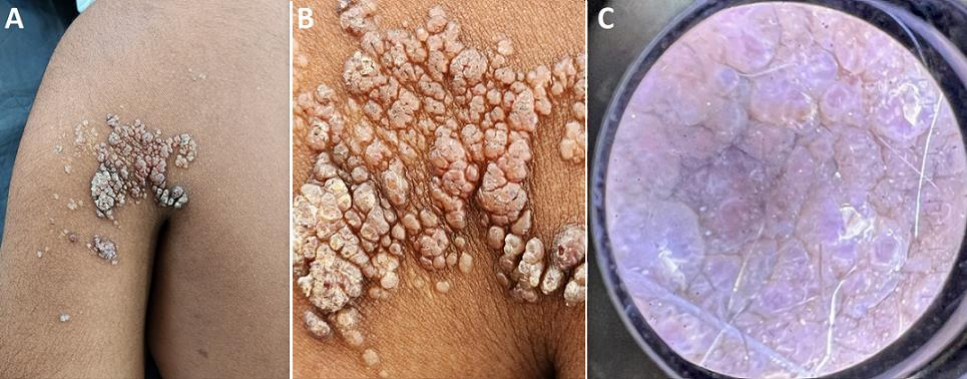
A) erythematous vesicles with crusts; B) clear, fluid filled vesicles; C) dermoscopy showing frogspawn like lesions with white lined structures

